# Circadian–immune crosstalk in insomnia disorder: mechanisms and therapeutic implications

**DOI:** 10.3389/fnins.2026.1881195

**Published:** 2026-07-14

**Authors:** Yini Huang, Xiuying Wang, Xiangjiao Chen, Yan Liu

**Affiliations:** Changsha Hospital of Traditional Chinese Medicine (Changsha Eighth Hospital), Changsha, China

**Keywords:** circadian rhythm, immune inflammation, insomnia disorder, low-grade inflammation, melatonin

## Abstract

Insomnia disorder (ID) is a common sleep–wake disorder characterized by persistent difficulty initiating or maintaining sleep, early-morning awakening, or non-restorative sleep, accompanied by daytime functional impairment. ID has traditionally been explained by the hyperarousal model, which emphasizes cognitive, emotional, cortical, neuroendocrine, and autonomic overactivation. However, this model alone does not fully account for the chronic persistence, relapse tendency, and multisystem associations of ID. Emerging evidence suggests that circadian rhythm disruption, impaired melatonin signaling, hypothalamic–pituitary–adrenal (HPA) axis activation, autonomic imbalance, and low-grade inflammation may also contribute to the development and maintenance of ID. Available evidence indicates that sleep disturbance is more consistently associated with selected inflammatory markers, particularly C-reactive protein (CRP) and interleukin-6 (IL-6), whereas findings for tumor necrosis factor-alpha (TNF-*α*) remain less consistent. The circadian system regulates sleep, endocrine function, metabolism, and immune-inflammatory activity through the suprachiasmatic nucleus, melatonin and cortisol rhythms, peripheral clock genes, and rhythmic immune-cell responses. Disruption of this temporal network may alter melatonin secretion, inflammatory rhythmicity, and stress-related neuroendocrine responses, thereby contributing to the persistence of insomnia symptoms. Compared with previous reviews that have separately discussed hyperarousal, circadian rhythm disruption, melatonin signaling, or sleep-related inflammation, this review integrates these processes into a circadian–immune perspective for understanding ID. We summarize alterations in sleep–wake rhythms, melatonin signaling, HPA-axis activity, autonomic regulation, and immune-inflammatory responses in ID, and discuss potential intervention strategies, including light management, melatonin and melatonin receptor agonists, cognitive behavioral therapy for insomnia (CBT-I), physical activity, time-restricted eating, and stress management. This review aims to provide a mechanistic basis for understanding the chronicity and heterogeneity of ID and for developing individualized intervention strategies.

## 1 Introduction

Insomnia disorder (ID) is one of the most common clinical sleep disorders and is typically characterized by persistent difficulty initiating sleep, difficulty maintaining sleep, early-morning awakening, or reduced restorative sleep, accompanied by daytime functional impairments such as fatigue, decreased attention, emotional instability, cognitive dysfunction, and reduced quality of life (Sutton, 2021; Paul and Salas, 2024). As a sleep–wake disorder explicitly recognized in the Diagnostic and Statistical Manual of Mental Disorders, Fifth Edition (DSM-5), International Classification of Sleep Disorders, Third Edition (ICSD-3), and International Classification of Diseases, Eleventh Revision (ICD-11), ID has a relatively standardized diagnostic framework. Its diagnosis mainly relies on clinical history taking and patients’ subjective reports of nocturnal sleep difficulties and daytime functional impairment (Sateia, 2014; Riemann et al., 2022). Epidemiological studies suggest that approximately 10% of adults meet diagnostic criteria for insomnia disorder, while about 20% of the population may occasionally experience insomnia symptoms, indicating a substantial population-level burden (Morin and Jarrin, 2022). Women, older adults, and individuals with socioeconomic difficulties may be at higher risk of insomnia disorder. In addition, insomnia may persist over time in a substantial subgroup of patients, although persistence estimates vary depending on the definition of insomnia, follow-up duration, and study cohort. Morin and Jarrin reported that approximately 40% of patients continued to experience insomnia symptoms over a 5-year follow-up period (Morin and Jarrin, 2022). Long-term insomnia not only impairs emotional regulation, cognitive performance, and social functioning, but is also closely associated with increased risks of depression, anxiety, and cardiometabolic diseases. Therefore, ID has become an important public health issue affecting both mental health and overall health status (Li et al., 2016; Javaheri and Redline, 2017).

At present, the treatment of insomnia disorder mainly includes non-pharmacological interventions and pharmacological therapy. Cognitive behavioral therapy for insomnia (CBT-I) is recommended by multiple guidelines as the first-line treatment for chronic insomnia disorder. It improves sleep–wake regulation primarily through sleep restriction, stimulus control, cognitive restructuring, and relaxation training (Qaseem et al., 2016; Edinger et al., 2021). Pharmacological treatment may be used for short-term symptom control or in patients with insufficient response to CBT-I. Commonly used medications include benzodiazepine receptor agonists, non-benzodiazepine hypnotics, melatonin receptor agonists, orexin receptor antagonists, and certain sedating antidepressants (Sateia et al., 2017; Riemann et al., 2023). However, current treatments still face several challenges, including limited accessibility and adherence to CBT-I, as well as restrictions on long-term medication use due to tolerance, dependence, and residual daytime effects. These limitations indicate the need to further elucidate the mechanisms underlying ID and to explore new therapeutic targets (Riemann et al., 2023).

Against this background, an in-depth understanding of the pathological mechanisms underlying ID is essential for optimizing therapeutic strategies. Traditionally, the onset and maintenance of ID have been closely linked to hyperarousal. Hyperarousal is not a single state of psychological tension, but rather a persistent activation across multiple domains, including cognitive, emotional, neuroendocrine, autonomic, and cortical processes. Specifically, patients may exhibit cognitive–emotional arousal, such as pre-sleep worry, rumination, and emotional tension, as well as physiological arousal characterized by hypothalamic–pituitary–adrenal (HPA)-axis activation, sympathetic nervous system excitation, increased cortisol secretion, and enhanced high-frequency electroencephalographic activity (Vgontzas et al., 2001; Riemann, 2010; Kalmbach et al., 2018b). These abnormal activations make it difficult for individuals to transition smoothly from wakefulness to stable sleep at night and may perpetuate daytime fatigue, abnormal vigilance, and impaired emotional regulation, thereby forming a vicious cycle of “sleep difficulty–daytime dysfunction–increased pre-sleep worry–further insomnia” (Riemann et al., 2010; Kalmbach et al., 2018a).

However, explaining ID solely through hyperarousal has certain limitations. Increasing evidence suggests that ID is not merely a consequence of elevated pre-sleep arousal, but may also involve an imbalance among sleep–wake rhythms, neuroendocrine rhythms, and immune-inflammatory rhythms (Riemann et al., 2010; Irwin, 2019; Zielinski and Gibbons, 2022). The circadian system, with the suprachiasmatic nucleus (SCN) as the central pacemaker, coordinates sleep–wake timing, neuroendocrine activity, and immune-inflammatory responses through melatonin secretion, cortisol rhythms, and peripheral clock gene networks (Dumbell et al., 2016; Fishbein et al., 2021). Circadian disruption may be associated with impaired sleep initiation and maintenance, stress-system activation, sympathetic excitation, and altered inflammatory rhythmicity (Balbo et al., 2010; Cermakian et al., 2014). Sleep insufficiency and insomnia-related chronic stress may also be associated with changes in selected inflammatory markers, particularly IL-6 and CRP, whereas findings for TNF-*α* remain less consistent. These processes may also be accompanied by alterations in immune-cell function, including monocytes, T cells, and natural killer cells, suggesting possible dysregulation of the neuroendocrine–immune network (Irwin, 2019; Besedovsky et al., 2019; Ren et al., 2026).

**Figure 1:**
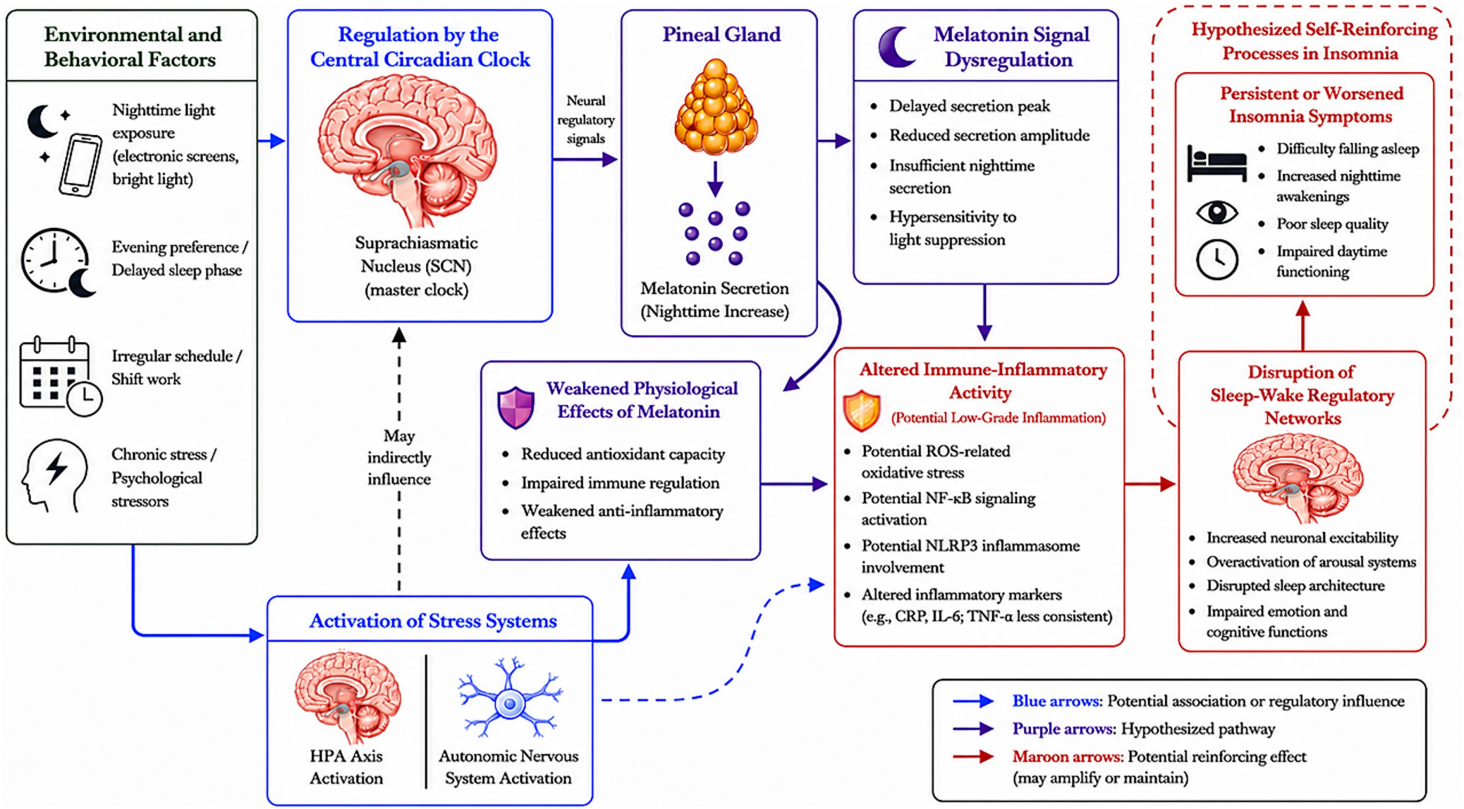
Hypothesized self-reinforcing circadian–immune–hyperarousal loop in insomnia disorder. This schematic illustrates a proposed model in which environmental and behavioral factors, including nighttime light exposure, delayed sleep phase, irregular schedules, shift work, and chronic psychological stress, may disturb central circadian regulation and activate stress-response systems. These changes may contribute to melatonin signal dysregulation, weakened physiological effects of melatonin, altered immune-inflammatory activity, and disruption of sleep–wake regulatory networks, thereby potentially contributing to persistent or worsened insomnia symptoms. In this model, hyperarousal, circadian misalignment, impaired melatonin signaling, HPA-axis/autonomic activation, and low-grade immune-inflammatory activity are conceptualized as interacting nodes within a hypothesized self-reinforcing process. Blue arrows indicate potential associations or regulatory influences, purple arrows indicate hypothesized pathways, and red arrows indicate potential reinforcing effects. This figure represents a conceptual synthesis based on current mechanistic, observational, and experimental evidence and does not imply a fixed causal sequence. Some pathways are supported mainly by preclinical studies or studies in non-insomnia populations and require further validation in patients with insomnia disorder.

Therefore, understanding ID from the perspective of circadian–immune interactions may help overcome the limitations of the traditional hyperarousal model in explaining the pathogenesis of insomnia. Building on previous authoritative reviews of sleep–immune interactions and circadian regulation, the present review further integrates the hyperarousal construct with circadian misalignment, weakened melatonin signaling, HPA-axis/autonomic activation, and low-grade immune inflammation. We propose that insomnia disorder may be better understood as a chronic and heterogeneous network condition in which these processes interact with one another and may contribute to symptom persistence. The novelty of this review lies in positioning the circadian–immune axis as a bridge between the classical hyperarousal model and emerging neuroimmune hypotheses, with particular emphasis on inflammatory rhythmicity rather than only static inflammatory marker levels. Accordingly, this review summarizes alterations in sleep–wake rhythms, melatonin signaling, stress-system activity, and immune-inflammatory responses in ID, aiming to provide a mechanistic reference for understanding its chronicity and for developing individualized intervention strategies. The hypothesized self-reinforcing circadian–immune–hyperarousal loop is summarized in Figure 1.

## 2 Methods and scope of the review

This article was designed as a narrative review rather than a systematic review or meta-analysis. Relevant literature was identified through searches of PubMed, Web of Science, and Google Scholar from database inception to May 2026. Search terms included combinations of “insomnia disorder,” “circadian rhythm,” “sleep–wake rhythm,” “melatonin,” “melatonin receptor agonist,” “HPA axis,” “autonomic nervous system,” “low-grade inflammation,” “immune inflammation,” “CRP,” “IL-6,” “TNF-*α*,” “IL-1β,” “clock genes,” “CBT-I,” “light therapy,” “chronotherapy,” “exercise,” and “time-restricted eating.” Priority was given to clinical guidelines, systematic reviews, meta-analyses, and human observational studies. Representative mechanistic and preclinical studies were also included when they were directly relevant to circadian–immune regulation in insomnia disorder. Additional references were identified from the reference lists of relevant articles. Because the aim of this review was to provide an integrative mechanistic synthesis rather than to estimate pooled effects, no formal risk-of-bias assessment, evidence grading, or quantitative meta-analysis was performed.

## 3 Circadian regulation of sleep

According to Borbély’s two-process model of sleep regulation, normal sleep is primarily regulated by the interaction between the sleep homeostatic process, also known as Process S, and the circadian process, also known as Process C (Borbély, 2022). Process S reflects the gradual accumulation of sleep pressure with increasing time spent awake and its progressive dissipation during sleep. Process C is mainly driven by the endogenous biological clock and determines sleep propensity, wakefulness, and alertness across the 24-h day. In simple terms, the sleep homeostatic process determines “how strong the sleep pressure is,” whereas the circadian process determines “when the body is most biologically prepared for sleep or wakefulness.” The coordinated interaction between these two processes enables individuals to fall asleep at an appropriate time and maintain wakefulness during the daytime. Therefore, the circadian system is not only the basis for sleep timing, but also an important regulatory network for maintaining sleep continuity, sleep quality, and multisystem physiological homeostasis (Deboer, 2018; Patke et al., 2020).

In mammals, circadian rhythms are primarily governed by the SCN, which serves as the central pacemaker and is located in the anterior hypothalamus. The SCN integrates environmental light signals and transmits temporal information to peripheral tissues, thereby coordinating multiple physiological processes, including the sleep–wake cycle, body temperature, hormone secretion, metabolic activity, and immune function (Reppert and Weaver, 2002; Hastings et al., 2018; Patke et al., 2020). In sleep regulation, the SCN coordinates the transition between sleep and wakefulness at appropriate times by regulating melatonin secretion, HPA-axis/cortisol rhythms, autonomic nervous system output, and the synchronization of peripheral clock genes (Doghramji, 2007; Hastings et al., 2018; O'Byrne et al., 2021). A dark nighttime environment promotes melatonin secretion by the pineal gland, decreases core body temperature, and attenuates the activity of wake-promoting systems, thereby facilitating sleep initiation (Cajochen et al., 2003; Zisapel, 2018). In contrast, increased morning light exposure suppresses melatonin secretion, elevates cortisol levels, and gradually promotes wakefulness (Wilhelm et al., 2007; O'Byrne et al., 2021). Accordingly, when circadian synchronization is disrupted by abnormal light exposure, irregular sleep–wake schedules, shift work, or delayed sleep phase, sleep initiation, sleep maintenance, and sleep quality may all be adversely affected (Dautovich et al., 2019).

At the molecular level, circadian rhythms are primarily maintained by transcription–translation feedback loops composed of core clock genes, including circadian locomotor output cycles kaput (CLOCK), brain and muscle ARNT-like 1 (BMAL1), period (PER), and cryptochrome (CRY) (Takahashi, 2016; Takahashi, 2017). In this system, CLOCK and BMAL1 form a transcriptional complex that promotes the expression of PER and CRY. After the accumulation of PER and CRY proteins, they in turn inhibit CLOCK/BMAL1 activity, thereby generating an approximately 24-h rhythmic oscillation (Takahashi, 2017). These core clock genes are not only present in the central SCN, but are also widely expressed in peripheral tissues, including the liver, adipose tissue, gastrointestinal tract, vascular endothelium, and immune cells (Mohawk et al., 2012; Schibler et al., 2015). Therefore, the circadian system does not merely regulate sleep timing, but also participates in a wide range of physiological processes, such as metabolism, endocrine function, immune regulation, and inflammatory responses (Patke et al., 2020). This feature suggests that circadian abnormalities in ID may not only affect sleep phase and sleep continuity, but may also alter the temporal distribution and intensity of immune-inflammatory responses through peripheral clock desynchronization.

## 4 Circadian regulation of immune function

The circadian system is one of the key factors influencing dynamic changes in immune function. Previous studies have shown that various immune cells, including monocytes, macrophages, neutrophils, T cells, B cells, and natural killer cells, express core clock genes such as CLOCK, BMAL1, PER, and CRY, and exhibit pronounced circadian rhythmicity in cell migration, proliferation, activation, and cytokine secretion (Cermakian et al., 2013; Labrecque and Cermakian, 2015; Scheiermann et al., 2018; Xiao et al., 2021). This rhythmic regulation enables the body to appropriately allocate immune surveillance, inflammatory responses, and tissue repair across different time periods, thereby maintaining immune homeostasis (Scheiermann et al., 2013; Besedovsky et al., 2019; Haspel et al., 2020). For example, the distribution of inflammatory cytokines, chemokines, and immune cells in peripheral blood and tissues is not constant, but is jointly regulated by central circadian signals, peripheral clock genes, the HPA axis, and the autonomic nervous system (Labrecque and Cermakian, 2015; Scheiermann et al., 2018). When sleep insufficiency, nocturnal light exposure, shift work, or circadian desynchronization disturbs this temporal regulatory network, rhythmic immune-cell activity may become altered and may be accompanied by changes in inflammatory markers such as IL-6 and CRP (Irwin, 2019; Garbarino et al., 2021; Irwin, 2023). However, these associations should not be interpreted as evidence that circadian disruption uniformly or directly drives chronic low-grade inflammation in all patients with ID. Rather, the circadian system may serve as an important regulatory context linking sleep disturbances, neuroendocrine stress, and immune-inflammatory abnormalities (Irwin and Opp, 2017).

For ID, circadian abnormalities are not limited to disruptions in sleep–wake timing, but may also be associated with changes in immune-cell function and inflammatory cytokine rhythmicity through alterations in melatonin secretion, HPA-axis activity, autonomic nervous system output, and peripheral clock-gene expression (Nader et al., 2010; Ding et al., 2024). Such disruption of “temporal architecture” may contribute to deviations from normal immune-inflammatory rhythmicity and may be related to a persistent, low-grade, non-infectious inflammatory state in some patients. Accordingly, circadian disruption should be regarded as a putative interacting factor linking insomnia symptoms, stress-system activation, and immune-inflammatory abnormalities, rather than as an independently established cause of immune dysregulation in ID.

It should also be noted that much of the evidence regarding circadian regulation of immune-cell trafficking, clock-gene expression, and cytokine rhythmicity comes from preclinical studies, experimental chronobiology, or studies in healthy human populations. Therefore, when these findings are applied to ID, they should be interpreted as mechanistic background rather than direct evidence that circadian disruption causes immune dysregulation in patients with ID. In addition, mechanistic pathways involving oxidative stress, NF-κB signaling, and NLRP3 inflammasome activation are supported mainly by preclinical or non-ID evidence and should be extrapolated to insomnia disorder with caution. Future studies in well-characterized ID populations with repeated sampling across the 24-h cycle are needed to validate these circadian–immune alterations.

## 5 Interactions between sleep disturbances and the immune system

Sleep is an important physiological basis for maintaining immune homeostasis and regulating inflammatory responses. Normal sleep helps optimize immune responses and maintain the dynamic balance of inflammation, whereas long-term sleep deprivation, sleep fragmentation, or reduced sleep quality may be associated with chronic low-grade inflammation and are closely associated with the occurrence and progression of metabolic abnormalities, cardiovascular diseases, and neurodegenerative disorders (Singh et al., 2025). Conversely, immune activation states, such as infection and inflammation, can also act on sleep-regulatory networks through cytokines and peripheral–central immune signaling, thereby altering sleep architecture and sleep–wake transitions. These findings suggest a bidirectional regulatory relationship between sleep and the immune system (Besedovsky et al., 2019).

When sleep disturbances persist, this bidirectional relationship may shift from a physiological homeostatic process toward a maladaptive cycle associated with symptom persistence. Long-term difficulty initiating sleep, difficulty maintaining sleep, sleep fragmentation, and circadian disruption may continuously weaken the restorative regulatory effects of sleep on the immune system, potentially increasing the likelihood of sustained inflammatory alterations. In turn, persistent inflammatory signals may act on central sleep–wake networks, further reducing sleep stability. Against this background, immune-inflammatory abnormalities have gradually become an important entry point for understanding the chronicity of ID (Garbarino et al., 2021).

Unlike the acute inflammatory responses commonly observed in infection or autoimmune diseases, immune alterations associated with ID are more likely to manifest as a mild, persistent, and non-infectious state of low-grade inflammation. This state is mainly characterized by slight elevations in peripheral inflammatory markers, reduced stability of inflammatory rhythms, and the continuous influence of peripheral inflammatory signals on central sleep–wake networks (Prather et al., 2015; Garbarino et al., 2021). Therefore, immune-inflammatory abnormalities in ID should not be simply understood as accompanying phenomena secondary to disturbed sleep. Instead, they may participate in the maintenance and prolongation of insomnia symptoms through a cyclic process of “sleep/circadian disruption–low-grade inflammatory activation–further impairment of sleep–wake regulation.”

### 5.1 Insomnia and peripheral inflammatory markers

Low-grade inflammation generally refers to a mild, persistent, and non-infectious state of inflammatory activation, which is commonly assessed using peripheral inflammatory markers such as CRP, IL-6, TNF-*α*, and interleukin-1 beta (IL-1β) (Del Giudice and Gangestad, 2018; Furman et al., 2019). Existing studies have shown that sleep disturbances, shortened sleep duration, and reduced sleep quality may all be associated with elevated peripheral inflammatory markers. A systematic review and meta-analysis by Irwin et al. (2016), which included 72 adult studies, showed that sleep disturbance was associated with increased levels of CRP and IL-6, while short sleep duration was mainly associated with elevated CRP but not significantly associated with IL-6.

Among these markers, CRP is a classic acute-phase reactant, primarily synthesized and released by hepatocytes in response to pro-inflammatory signals such as IL-6 and IL-1β. Its serum level can increase rapidly during systemic inflammatory responses, reflecting the peripheral inflammatory burden and chronic inflammatory status (Pepys and Hirschfield, 2003; Sproston and Ashworth, 2018). IL-6 is not only an important pro-inflammatory cytokine, but is also considered to be involved in the regulation of sleep–wake processes and circadian rhythms. Previous studies have shown that IL-6 exhibits a circadian secretion rhythm in humans, with levels typically increasing at night, and is associated with sleep propensity, sleep depth, and changes in sleep architecture. Partial sleep deprivation can alter the nocturnal secretion pattern of IL-6, suggesting that IL-6 may serve as an important mediator linking disrupted sleep architecture, increased wakefulness, and peripheral inflammatory responses (Vgontzas et al., 1999; Redwine et al., 2000; Vgontzas et al., 2005).

Notably, this meta-analysis did not identify a consistent association between sleep disturbance or sleep duration and TNF-*α*. In addition, experimental sleep deprivation or sleep restriction did not exert significant effects on CRP, IL-6, or TNF-α, suggesting that different inflammatory markers may vary in their sensitivity to sleep disruption (Irwin et al., 2016). Thus, there is an association between sleep disturbance and chronic low-grade inflammation, rather than a simple relationship in which “sleep insufficiency universally increases all inflammatory cytokines.” This feature also suggests that studies of ID should integrate peripheral markers such as CRP and IL-6, as well as their circadian rhythmic changes, to further clarify the role of sleep–immune interactions in disease chronicity.

### 5.2 Effects of inflammatory signals on sleep–wake regulation

Peripheral inflammatory signals may influence the central nervous system through multiple pathways, including vagal afferent signaling, alterations in blood–brain barrier permeability, signal transduction via cerebrovascular endothelial cells, sensing by circumventricular organs, and activation of microglia and astrocytes. After entering or acting on the central nervous system, inflammatory signals may affect brain regions involved in sleep, emotion, and cognitive regulation, including the hypothalamus, brainstem arousal systems, limbic system, and prefrontal cortex (Dantzer et al., 2000; Quan and Banks, 2007; Banks, 2015). These central effects may alter the threshold for sleep–wake transitions, sleep architecture, and neural network excitability, making individuals more prone to increased nocturnal awakenings, reduced sleep continuity, and impaired subjective sleep quality.

In addition, inflammatory signals may indirectly exacerbate insomnia by affecting emotional and cognitive systems. Patients with ID often experience pre-sleep worry, rumination, anxiety tendencies, and daytime cognitive fatigue (Riemann et al., 2010). Pro-inflammatory cytokines may enhance negative emotional processing and cognitive–emotional arousal by influencing monoamine neurotransmitter metabolism, the tryptophan–kynurenine pathway, glutamatergic transmission, HPA-axis feedback, and prefrontal–limbic functional connectivity, thereby aggravating difficulty initiating sleep, difficulty maintaining sleep, and reduced subjective sleep quality (Dantzer et al., 2008; Miller and Raison, 2016; Haroon et al., 2017). Therefore, the effects of inflammatory signals on insomnia are not limited to direct interference with sleep-regulatory networks; they may also contribute to the maintenance of insomnia symptoms by intensifying emotional–cognitive hyperarousal.

### 5.3 Bidirectional cycle between insomnia and low-grade inflammation

There may be a mutually reinforcing and progressively amplified pathological relationship between ID and low-grade inflammation. Inflammatory molecules can regulate sleep and the circadian system, while changes in sleep and circadian rhythms can, in turn, modulate inflammatory processes (Besedovsky et al., 2019; Zielinski and Gibbons, 2022; Veler, 2023). Specifically, shortened sleep duration, sleep fragmentation, and reduced sleep quality may weaken the role of sleep in maintaining immune homeostasis, making the body more susceptible to elevated peripheral inflammatory markers, such as CRP and IL-6, as well as disrupted inflammatory rhythms (Irwin et al., 2016). Conversely, persistent low-grade inflammation may affect sleep–wake networks through peripheral–central immune signaling and further aggravate difficulty initiating sleep, difficulty maintaining sleep, and reduced subjective sleep quality by enhancing emotional–cognitive hyperarousal (Dantzer et al., 2008).

This bidirectional cycle may help explain why insomnia symptoms persist or recur in some patients rather than resolving after a short-term sleep disturbance. In some patients, low-grade inflammation may not merely be an accompanying phenomenon, but rather one biological factor associated with the maintenance and recurrence of insomnia (Irwin et al., 2016; Irwin and Opp, 2017). When patients remain in this cycle over the long term, they may not only experience nocturnal symptoms such as difficulty initiating sleep, increased awakenings, and reduced restorative sleep, but also present with daytime fatigue, mood fluctuations, anxiety or depressive tendencies, and cognitive decline (Fortier-Brochu et al., 2012; Bickley et al., 2021). Therefore, when understanding the chronicity of insomnia, it is necessary to integrate sleep rhythm disruption, low-grade inflammation, and emotional–cognitive hyperarousal into an interconnected network rather than treating them as isolated mechanisms.

## 6 Melatonin signaling: a key bridge linking circadian rhythms and immune inflammation

### 6.1 Melatonin and sleep phase regulation

Melatonin is an indoleamine hormone secreted mainly at night by the pineal gland, and its synthesis and release are jointly regulated by environmental light exposure and the central biological clock in the SCN (Brown, 1994; Arendt and Aulinas, 2022; Ackermann and Stehle, 2006). Studies have shown that melatonin can synchronize circadian rhythms and improve sleep initiation, sleep duration, and sleep quality (Cajochen et al., 2003). It also plays a central role in antioxidative defense, circadian rhythm maintenance, sleep regulation, and neuronal survival (Xie et al., 2017). Under normal physiological conditions, melatonin levels rise at night, signaling the onset of the biological night. After morning light exposure increases, melatonin secretion is suppressed, and wakefulness is enhanced (Zisapel, 2018; de Toledo et al., 2023). In ID, abnormalities in melatonin signaling may manifest as a delayed secretion peak, reduced secretion amplitude, insufficient nocturnal secretion, or excessive sensitivity to light-induced suppression (Poza et al., 2022). For patients mainly characterized by difficulty initiating sleep, a tendency toward late sleep timing, or delayed sleep phase, abnormal melatonin rhythms may represent an important mechanism (Zisapel, 2018). Nighttime use of electronic screens, exposure to bright light, or long-term irregular sleep–wake schedules may suppress or delay melatonin secretion, leaving patients in a state of relatively high wakefulness at conventional bedtime (Gooley et al., 2011; Chang et al., 2015).

### 6.2 Immunomodulatory and anti-inflammatory effects of melatonin

In addition to its role in circadian and sleep–wake regulation, melatonin also exerts immunomodulatory, antioxidative, and anti-inflammatory effects. It may attenuate inflammatory responses by directly scavenging reactive oxygen species, enhancing antioxidative defense, inhibiting nuclear factor-kappa B (NF-κB)-mediated inflammatory signaling, regulating NOD-like receptor family pyrin domain-containing 3 (NLRP3) inflammasome activation, and reducing the release of pro-inflammatory cytokines such as IL-1β, IL-6, and TNF-*α* (Reiter et al., 2016; Favero et al., 2017; Hardeland, 2018; Arioz et al., 2021). However, these molecular mechanisms are supported mainly by preclinical studies or studies in non-insomnia populations and should therefore be extrapolated to ID with caution. For example, a systematic review and meta-analysis of clinical trials by Cho et al. showed that exogenous melatonin reduced the levels of several inflammatory markers, including interleukin-1 (IL-1), IL-6, and interleukin-8 (IL-8), whereas its effect on TNF was inconsistent (Cho et al., 2021). These findings suggest that melatonin may serve as an important molecular bridge linking circadian rhythms and inflammatory responses. However, it should be noted that much of the evidence supporting the anti-inflammatory effects of melatonin comes from studies in metabolic diseases, inflammatory disorders, or other clinical populations, and therefore cannot be directly generalized to patients with ID. In the context of ID, melatonin is more appropriately understood as a mechanistic node connecting circadian imbalance and inflammatory abnormalities, rather than being simply defined as a routine anti-inflammatory agent for insomnia-related inflammation. In other words, the value of melatonin in ID should first be interpreted in terms of circadian regulation, while its potential anti-inflammatory effects may represent additional or indirect consequences of circadian realignment.

### 6.3 Potential mechanisms by which the melatonin–inflammation axis contributes to the maintenance of insomnia

The melatonin–inflammation axis may represent an important mechanistic link connecting circadian disruption, immune-inflammatory abnormalities, and the chronicity of ID. In some patients with insomnia, nocturnal light exposure, a tendency toward late sleep timing, delayed sleep phase, and irregular sleep–wake schedules may be associated with a delayed phase of melatonin secretion, reduced nocturnal secretion amplitude, or weakened biological night signals, which may leave the body in a relatively heightened state of wakefulness at conventional bedtime. Previous studies have suggested that sleep-onset insomnia is often associated with a later circadian phase, and circadian delay may increase repeated experiences of failed sleep initiation, potentially contributing to the development and maintenance of conditioned insomnia (Nobre et al., 2021; Lack et al., 2023). Thus, abnormalities in melatonin signaling may not only affect sleep initiation, but also increase the instability of sleep–wake transitions by weakening circadian synchronization.

From the perspective of immune inflammation, weakened melatonin signaling may further impair nocturnal antioxidative and anti-inflammatory regulation. Melatonin itself can scavenge reactive oxygen species, stabilize mitochondrial function, inhibit NF-κB-mediated inflammatory signaling, regulate NLRP3 inflammasome activation, and reduce the release of pro-inflammatory cytokines (Reiter et al., 2016; Favero et al., 2017; Arioz et al., 2021). When nocturnal melatonin secretion is insufficient or its rhythmic phase is disrupted, the rhythmic suppression of oxidative stress and inflammatory responses may be weakened, making low-grade inflammation more likely to persist. Meanwhile, long-term insomnia, sleep insufficiency, and chronic psychological stress can activate the HPA axis and sympathetic nervous system, promote inflammatory cytokine release, and further aggravate neuroendocrine and immune rhythm dysregulation (Nicolaides et al., 2020; Irwin, 2019). These findings suggest that melatonin abnormalities may represent one component of the biological context associated with chronic insomnia, with stress-system activation, increased oxidative stress, and the development of low-grade inflammation.

More importantly, inflammatory responses may in turn interfere with sleep–wake regulation. Persistent low-grade inflammation can affect the hypothalamus, brainstem arousal systems, glial cells, and brain regions involved in emotional and cognitive regulation through peripheral–central immune signaling, thereby altering neural excitability and sleep architecture while enhancing anxiety, rumination, and cognitive–emotional hyperarousal (Dantzer et al., 2008; McCusker and Kelley, 2013; Amini et al., 2022). Therefore, patients with insomnia may be involved in a hypothesized cycle of “circadian misalignment–weakened melatonin signaling–reduced anti-inflammatory/antioxidative regulation–enhanced low-grade inflammation–further disturbance of sleep–wake networks.” This cycle may help explain why some patients experience worsening difficulty initiating sleep, increased nocturnal awakenings, and reduced restorative sleep under conditions of long-term irregular sleep–wake schedules, nocturnal light exposure, or chronic stress.

## 7 Intervention implications based on the circadian–immune axis

Based on the circadian–immune framework discussed above, several intervention strategies may be considered for insomnia disorder, including light management, melatonin and melatonin receptor agonists, CBT-I, physical activity, scheduled eating, and stress management. These approaches do not directly target inflammation as standard anti-inflammatory therapies, but may help stabilize sleep–wake rhythms, improve circadian synchronization, reduce hyperarousal, and indirectly modulate neuroendocrine–immune homeostasis. The main intervention strategies, target processes, potential mechanisms, and applicable populations are summarized in Table 1.

**Table 1:** Insomnia intervention strategies based on the circadian–immune axis.

Intervention strategy	Main target process	Potential mechanism	Applicable population	References
Morning light exposure	Circadian realignment	Advances sleep phase and stabilizes melatonin rhythms	Individuals with late sleep timing, difficulty initiating sleep, or delayed circadian phase	(Czeisler and Gooley, 2007; Tähkämö et al., 2019)
Reduction of nighttime blue-light exposure	Melatonin preservation	Prevents light-induced suppression of melatonin secretion and circadian phase delay at night	Individuals who use electronic screens before bedtime	(Chang et al., 2015; Faulkner et al., 2019)
Melatonin / melatonin receptor agonists	Sleep phase regulation	Provides a biological nighttime signal and improves circadian misalignment; prolonged-release melatonin may be used in some older adults with insomnia	Individuals with delayed sleep phase or older adults with insomnia	(Ferracioli-Oda et al., 2013; Auld et al., 2017)
CBT-I	Behavioral rhythm stabilization	Reduces pre-sleep arousal and stabilizes wake-up time and sleep pressure through sleep restriction, stimulus control, and cognitive restructuring	Patients with chronic insomnia	(Morin et al., 2006; Edinger et al., 2021)
Regular physical activity	Circadian and inflammatory regulation	Enhances daytime activity levels, increases sleep drive, improves emotional status, and may modulate inflammatory markers	Individuals with comorbid anxiety, metabolic abnormalities, or sedentary behavior	(Xie et al., 2021; Rubio-Valles and Ramos-Jimenez, 2025)
Scheduled eating	Peripheral clock synchronization	Acts as a synchronizing cue for peripheral clocks, stabilizes metabolic rhythms, and reduces nighttime metabolic burden	Individuals with irregular sleep–wake schedules or eating patterns	(Wehrens et al., 2017; BaHammam and Pirzada, 2023)
Stress management	HPA-axis regulation	Reduces chronic stress-related sustained activation of the HPA axis and sympathetic nervous system, and alleviates cognitive–emotional hyperarousal	Individuals with high stress levels or prominent rumination	(Hirotsu et al., 2015; Kalmbach et al., 2018a)

### 7.1 Light management and circadian realignment

Light is the most important external cue for regulating circadian rhythms (Takahashi, 2017). Morning light exposure helps advance sleep phase, enhance daytime wakefulness, and stabilize nocturnal melatonin rhythms (Crowley and Eastman, 2015). In contrast, exposure to bright light at night, particularly blue light, may suppress melatonin secretion, delay sleep phase, and aggravate difficulty initiating sleep (Gooley et al., 2011; Silvani et al., 2022). Therefore, reducing electronic screen use before bedtime, lowering nighttime light intensity, and increasing exposure to natural morning light are important foundational strategies for improving insomnia associated with circadian disruption (Blume et al., 2019; Sun and Chen, 2022). From the perspective of circadian–immune interactions, light management may not only improve sleep onset timing, but may also indirectly influence inflammatory status by restoring melatonin rhythms, reducing nocturnal awakenings, and improving autonomic nervous system balance (Blume et al., 2019; Sun and Chen, 2022). The European insomnia guideline indicates that light therapy may serve as an adjunctive intervention to CBT-I (Riemann et al., 2023). The clinical value of light management lies primarily in restoring circadian synchronization and improving sleep onset timing and sleep stability. For patients with delayed sleep phase, excessive nocturnal light exposure, or insufficient morning light exposure, individualized light-based interventions may help improve sleep onset timing and sleep stability.

### 7.2 Melatonin and melatonin receptor agonists

The role of melatonin in the treatment of insomnia should be understood as circadian regulation rather than simply as sedative–hypnotic action (Zisapel, 2018). For patients with delayed sleep phase, circadian misalignment, or insomnia associated with age-related reductions in melatonin secretion, melatonin or melatonin receptor agonists may have potential therapeutic value (Lemoine et al., 2007; Burgess and Emens, 2016; Riemann et al., 2023). However, the key to melatonin treatment lies in the timing of administration, rather than the dose alone. If administered at an inappropriate time, melatonin may fail to achieve the intended adjustment of sleep phase and may even weaken its circadian realignment effect (Lewy et al., 2005; Riemann et al., 2023). Although systematic reviews of clinical trials have suggested that melatonin may reduce the levels of certain inflammatory cytokines, such as IL-1, IL-6, and IL-8 (Cho et al., 2021), current insomnia treatment guidelines mainly position melatonin as an adjunctive treatment for specific populations or circadian-related insomnia, rather than as a routine intervention targeting inflammatory pathways. At present, melatonin cannot be recommended as a standard anti-inflammatory treatment for patients with insomnia (Dujardin et al., 2020; Riemann et al., 2023). In both clinical practice and research, melatonin may be regarded as an important tool for regulating sleep phase and circadian synchronization, but its potential immunomodulatory effects still need to be further validated in populations with insomnia.

**Figure 2:**
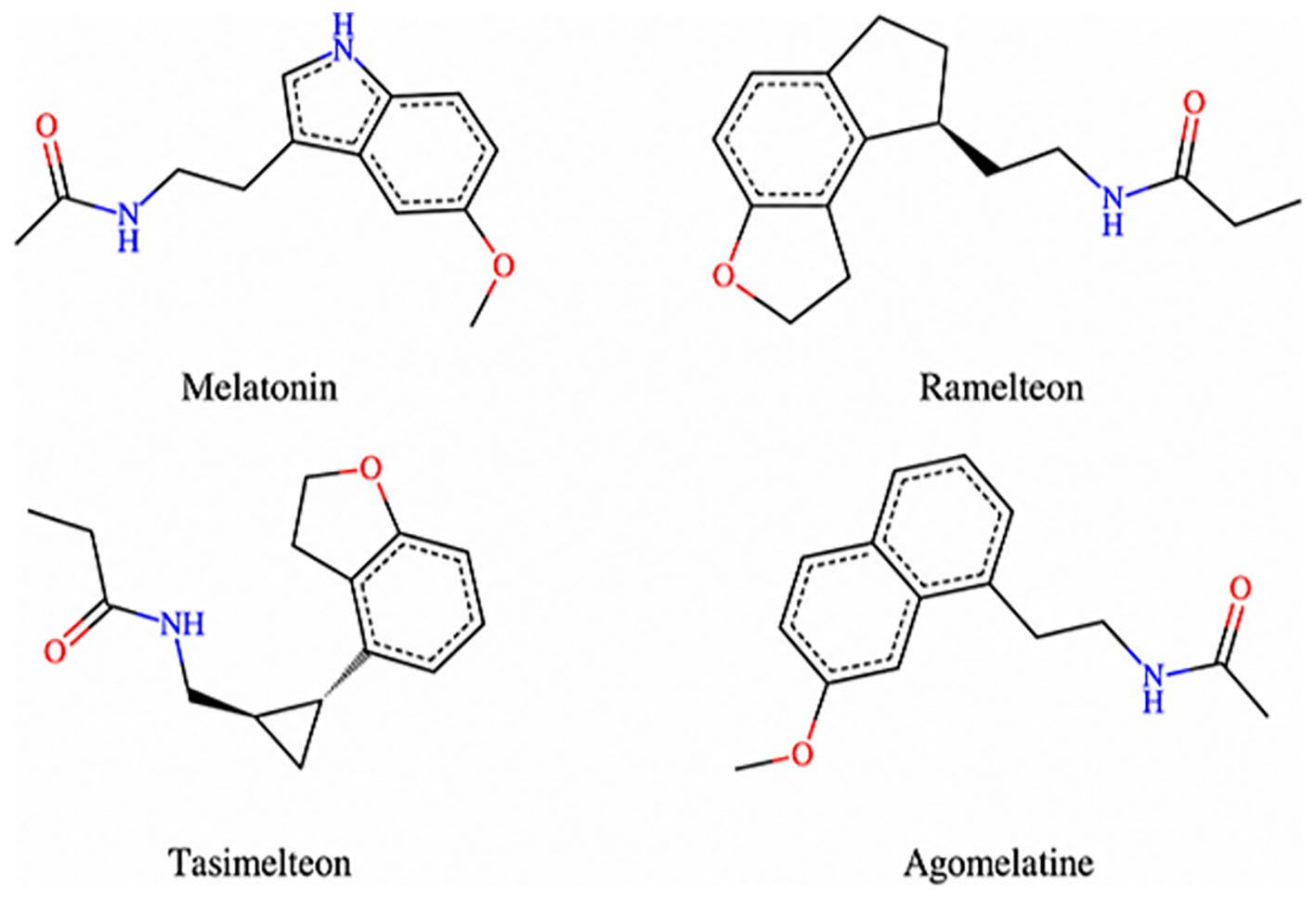
Chemical structures of melatonin and representative melatonin receptor agonists. This figure presents the chemical structures of melatonin and representative melatonergic agents, including ramelteon, tasimelteon, and agomelatine. These compounds are mainly discussed in relation to circadian phase regulation and melatonin receptor-mediated sleep–wake modulation, rather than as direct anti-inflammatory therapies for insomnia disorder.

More recent evidence has further refined the clinical positioning of melatonin and melatonergic agents in insomnia management. Prolonged-release melatonin has been discussed as a chronopharmaceutical strategy for restoring sleep–wake rhythms in selected patients with insomnia disorder, especially in the context of circadian medicine (Del Casale et al., 2024). In addition, the therapeutic effects of melatonin are influenced not only by dose, but also by the timing of administration relative to the individual sleep episode and circadian phase (Cruz-Sanabria et al., 2024). Melatonergic receptor agonists, including ramelteon, tasimelteon, and agomelatine, also differ in receptor affinity, pharmacokinetic properties, approved indications, and clinical applicability (Żełabowski et al., 2025). Ramelteon is mainly used for insomnia characterized by sleep-onset difficulty, whereas tasimelteon is more specifically associated with circadian rhythm sleep–wake disorders, particularly non-24-h sleep–wake disorder. Agomelatine has melatonergic receptor agonist activity, but its clinical use is more commonly discussed in relation to mood disorders (Żełabowski et al., 2025). Therefore, melatonin and melatonergic receptor agonists should not be regarded as a homogeneous class of sedative or anti-inflammatory agents; rather, their clinical interpretation should take into account patient phenotype, circadian phase, timing of administration, and comorbid conditions (Cruz-Sanabria et al., 2024; Żełabowski et al., 2025). The chemical structures of melatonin and representative melatonin receptor agonists are shown in Figure 2.

### 7.3 CBT-I and behavioral rhythm stabilization

CBT-I is the first-line treatment for chronic insomnia (Asnis et al., 2015; Edinger et al., 2021). Its core components include stimulus control, sleep restriction, cognitive restructuring, relaxation training, and sleep hygiene education (Walker et al., 2022). Although CBT-I is usually regarded as a psychological and behavioral therapy, its effects are not limited to improving sleep-related cognition (Altena et al., 2023). A fixed wake-up time, reduced time spent awake in bed, restriction of daytime napping, and the establishment of a stable sleep–wake rhythm can all help restore sleep homeostasis and circadian rhythms (Maurer et al., 2018). From the perspective of circadian–immune interactions, CBT-I may stabilize behavioral rhythms by improving sleep continuity, reducing pre-sleep arousal, and attenuating stress responses (Irwin et al., 2014; Thakral et al., 2020; Palagini et al., 2022). For example, sleep restriction and stimulus control can reduce time spent awake in bed and weaken the conditioned association between the bed and wakefulness-related experiences (Morin et al., 2006). Cognitive restructuring and relaxation training can alleviate pre-sleep worry, anxiety, and rumination, thereby reducing cognitive–emotional hyperarousal (van Straten et al., 2018). These changes may indirectly influence inflammatory status by improving sleep continuity and regulating HPA-axis and autonomic nervous system activity (Irwin et al., 2014; Redeker et al., 2020). Therefore, CBT-I should be understood primarily as a behavioral and rhythm-stabilizing intervention, rather than as a direct anti-inflammatory treatment (Irwin et al., 2015).

### 7.4 Lifestyle rhythms and anti-inflammatory management

Regular physical activity, scheduled eating, reduced nighttime food intake, weight management, and stress management can all serve as lifestyle-level rhythm-regulating factors that influence circadian stability, metabolic homeostasis, and inflammatory status (Chang and Su, 2025). Among these factors, physical activity may promote the stabilization of sleep–wake rhythms by enhancing daytime activity levels, increasing sleep drive, improving emotional status, and modulating inflammatory markers (Shen et al., 2023). Regular eating, in turn, can act as an important synchronizing signal for peripheral biological clocks, thereby helping maintain metabolic rhythms and energy homeostasis (Reytor-González et al., 2025). In contrast, nighttime eating, sedentary behavior, physical inactivity, and long-term psychological stress may further increase the burden of low-grade inflammation by aggravating circadian misalignment, metabolic dysregulation, and stress-axis activation (Kuang et al., 2018; Kim et al., 2025; Wu et al., 2025). Therefore, the value of lifestyle interventions in insomnia management should not be understood merely as general health advice, but rather as important adjunctive strategies for stabilizing circadian rhythms and improving neuroendocrine–immune homeostasis. For patients with insomnia who also have metabolic abnormalities, obesity, chronic stress, or a sedentary lifestyle, regular physical activity, scheduled eating, and stress management may provide more pronounced comprehensive benefits. However, current insomnia treatment guidelines still recommend CBT-I as the first-line treatment for chronic insomnia and limit pharmacological therapy to the short-term or individualized use of specific insomnia medications. Anti-inflammatory drugs or immunosuppressive therapies have not been incorporated into standard treatment regimens for insomnia and, therefore, should not be used as routine therapeutic strategies for insomnia (Edinger et al., 2021; Buysse et al., 2026).

## 8 Discussion

ID has long been interpreted within the framework of hyperarousal. This model proposes that patients remain in a state of sustained activation across cognitive, emotional, cortical, HPA-axis, and sympathetic domains, thereby contributing to difficulty initiating sleep, difficulty maintaining sleep, and daytime functional impairment (Riemann et al., 2010; Riemann, 2010; Van Someren, 2021). Although this model remains important for explaining the onset and maintenance of ID, it is insufficient to fully account for the prolonged disease course, recurrent relapse, and frequent comorbidity with chronic conditions such as mood disorders, metabolic abnormalities, and cardiovascular diseases in some patients. Based on the available evidence, circadian disruption, altered melatonin signaling, neuroendocrine stress, and low-grade immune-inflammatory responses may jointly contribute to the chronicity of ID (Irwin and Opp, 2017; Irwin, 2019; Feuth, 2024). Therefore, circadian–immune interactions may be regarded as a complementary extension of the classical hyperarousal model rather than a replacement for it. Previous reviews have established the close relationship between sleep and immune regulation, particularly the effects of sleep disturbance on inflammatory activity. Building on this literature, the present review focuses more specifically on ID as a chronic and heterogeneous clinical condition. Rather than treating hyperarousal, circadian misalignment, melatonin signaling, and immune inflammation as separate mechanisms, we discuss how these processes may converge within a shared circadian–immune framework.

In terms of evidence strength, relatively consistent findings can be summarized in two major aspects. First, the circadian system coordinates melatonin secretion, cortisol rhythms, autonomic activity, peripheral clock-gene expression, immune-cell trafficking, and cytokine secretion (Scheiermann et al., 2013; Partch et al., 2014; Borbély, 2022; Ding et al., 2024), providing a biological basis for the interaction between circadian regulation and immune function in ID. Second, systematic reviews and meta-analyses have shown that sleep disturbance is more consistently associated with changes in CRP and IL-6, whereas the association between TNF-*α* and sleep disturbance remains inconsistent (Irwin et al., 2016). Thus, insomnia-related inflammation should not be simply described as a generalized elevation of all pro-inflammatory markers, but may instead reflect low-grade inflammatory alterations with marker specificity and interindividual heterogeneity. Moreover, because immune-inflammatory activity itself exhibits circadian rhythmicity, single-time-point measurements may fail to capture changes in inflammatory phase, amplitude, and temporal stability. Future studies should therefore pay greater attention to inflammatory rhythmicity rather than relying solely on static inflammatory levels.

Although peripheral inflammatory signals may participate in the regulation of central sleep–wake and emotion-related networks (Krueger et al., 2001; Dantzer et al., 2008; Irwin, 2019; Zielinski and Gibbons, 2022), current evidence does not establish a clear unidirectional causal pathway in patients with ID. Based on the available evidence, low-grade inflammation is more appropriately interpreted as a process intertwined with sleep insufficiency, circadian misalignment, emotional stress, and HPA-axis activation, rather than as an independent cause of ID. This interpretation may also help explain the inconsistent inflammatory findings reported across different ID populations. Melatonin similarly illustrates the boundaries of current mechanistic interpretation. It is closely related to circadian phase regulation and also has antioxidative and immunomodulatory properties (Carrillo-Vico et al., 2013; Cho et al., 2021). However, clinical evidence supporting an anti-inflammatory role of melatonin in ID remains insufficient, and much of the available evidence derives from metabolic, inflammatory, or other non-insomnia populations. Therefore, in the treatment of insomnia, melatonin should primarily be positioned as a circadian-regulating signal rather than as a routine anti-inflammatory therapeutic strategy (Hamel and Horton, 2022).

This perspective also helps clarify the role of existing interventions. CBT-I, light management, melatonin or melatonergic agents, regular physical activity, scheduled eating, and stress management may all be relevant to circadian–immune regulation (Morin et al., 2006; Irwin et al., 2015; Tähkämö et al., 2019; Redeker et al., 2020; Edinger et al., 2021; Arnold, 2022; Shen et al., 2023; Reytor-González et al., 2025). However, their effects are more likely to be mediated through improved sleep continuity, stabilization of behavioral rhythms, reduction of cognitive–emotional hyperarousal, and attenuation of sustained stress-system activation. These strategies should therefore be understood as rhythm-stabilizing and hyperarousal-reducing interventions rather than direct anti-inflammatory treatments. In this context, time-based therapeutic concepts in traditional Chinese medicine (TCM), particularly timing acupuncture, may provide an additional exploratory perspective (Samuels, 2000; Wu and Zhao, 2024). A recent review summarized clinical and *in vivo* evidence suggesting that acupuncture may influence sleep-related circadian rhythms in circadian rhythm sleep–wake disorders, and discussed potential mechanisms involving central and peripheral biological clocks and neurochemical regulation (Wu and Zhao, 2024). These concepts are broadly consistent with the circadian-oriented perspective of the present review. In addition, TCM-based approaches, including acupuncture and herbal medicine, have been reviewed in relation to the neurobiological mechanisms of insomnia (Wang et al., 2023). Nevertheless, direct evidence linking TCM-based time interventions to circadian phase, inflammatory rhythmicity, and insomnia outcomes remains limited. Therefore, these interventions should be regarded as a low-certainty and exploratory area requiring further high-quality clinical studies.

Although circadian–immune interactions provide a relatively integrated theoretical framework for understanding ID, several limitations remain. First, ID is highly heterogeneous. Different patients may present with different phenotypes, such as difficulty initiating sleep, difficulty maintaining sleep, early-morning awakening, reduced subjective sleep quality, or delayed sleep phase, and their rhythm abnormalities and inflammatory profiles may not be consistent. Second, most existing studies have used cross-sectional designs and single-time-point measurements of inflammatory markers, making it difficult to determine the temporal sequence and causal relationships among circadian disruption, melatonin abnormalities, HPA-axis activation, and increased inflammation. Third, patients with ID often have comorbid anxiety, depression, obesity, metabolic syndrome, or chronic pain, all of which may themselves influence inflammatory levels and sleep quality, increasing the complexity of mechanistic interpretation. Finally, high-quality clinical studies remain relatively limited regarding whether CBT-I, light therapy, melatonin, physical activity, and scheduled eating can simultaneously improve sleep outcomes, circadian indicators, and inflammatory status.

Future research should place greater emphasis on mechanistic subtyping and dynamic assessment of ID. First, patients with ID can be classified according to clinical phenotypes, such as sleep-onset insomnia, sleep-maintenance insomnia, early-morning awakening, delayed sleep phase, and objective short sleep duration, to compare whether they differ in melatonin rhythms, cortisol rhythms, and inflammatory characteristics. Second, multi-time-point sampling strategies should be adopted to dynamically assess melatonin, cortisol, CRP, IL-6, TNF-*α*, and immune cell subsets, so as to reveal alterations in inflammatory rhythms rather than merely changes in inflammatory levels in ID. Third, multidimensional biomarker models should be established by integrating polysomnography, actigraphy, dim-light melatonin onset (DLMO), sleep questionnaires, psychological stress assessment, and inflammatory markers. Fourth, mechanistic intervention studies should be conducted to determine whether CBT-I, morning light exposure, melatonin, physical activity, scheduled eating, and stress management can simultaneously improve sleep, circadian rhythms, and inflammatory networks. Through these studies, the management of ID may gradually shift from symptom-oriented treatment toward mechanism-based and individualized intervention.

In summary, circadian–immune interactions may serve as an important complementary framework for explaining the chronicity of ID. This framework emphasizes that ID is not a single sleep problem, but rather the result of interactions among sleep–wake rhythms, melatonin signaling, HPA-axis/sympathetic activity, and immune-inflammatory responses. Compared with focusing solely on sleep duration or individual inflammatory markers, future research should pay greater attention to circadian synchronization, inflammatory rhythmicity, and the overall state of the neuroendocrine–immune network. Based on this perspective, stabilizing circadian rhythms, improving sleep continuity, reducing hyperarousal, and optimizing lifestyle patterns may become important directions for the comprehensive management of ID.
